# Response of riparian vegetation to short‐ and long‐term hydrologic variation

**DOI:** 10.1002/eap.2689

**Published:** 2022-08-07

**Authors:** Jonathan M. Friedman, Abigail M. Eurich, Gregor T. Auble, Michael L. Scott, Patrick B. Shafroth, Polly P. Gibson

**Affiliations:** ^1^ U.S. Geological Survey Fort Collins Science Center Fort Collins Colorado USA; ^2^ Under Contract to U.S. Geological Survey Fort Collins Science Center Fort Collins Colorado USA

**Keywords:** competition, desiccation, disturbance, flood, flow regulation, functional traits, inundation duration, logistic regression, reservoir, response time, species richness

## Abstract

Increasing demand for river water now conflicts with an increasing desire to maintain riparian ecosystems. Efficiently managing river flows for riparian vegetation requires an understanding of the time scale of flow effects, but this information is limited by the absence of long‐term studies of vegetation change in response to flow variation. To investigate the influence of short‐ and long‐term flow variability and dam operation on riparian vegetation, we determined the occurrence of 107 plant species in 133 permanent plots of known inundating discharge along the Gunnison River in Colorado on five different occasions between 1990 and 2013. Individual species moved up and down the gradient of inundating discharge coincident with increases and decreases in mean annual flow, and the correlations between flow and species occurrence were strongest when flows were weighted by time before vegetation sampling with a median half‐life of 1.5 years. Some tall, rhizomatous, perennial species, however, responded to flows on a longer time scale. Logistic regression of species occurrence showed a significant relation with inundation duration for 70 out of 107 species. Plot species richness and total vegetative cover decreased in association with desiccation at low inundation durations and with fluvial disturbance at high inundation durations. Within‐plot similarity in species occurrence between years decreased strongly with increasing inundation duration. Moderate inundation durations were dominated by tall, rhizomatous, perennial herbs, including invasive *Phalaris arundinacea* (reed canary grass). Over the 23‐year study period, species richness declined, and the proportion of rhizomatous perennials increased, consistent with the hypothesis that decreases in flow peaks and increases in low flows caused by flow regulation have decreased establishment opportunities for disturbance‐dependent species. In summary, annual‐scale changes in vegetation were strongly influenced by flow variation, and decadal‐scale changes were influenced by decreases in fluvial disturbance from upstream flow regulation beginning decades prior to the onset of this study.

## INTRODUCTION

Regulation of the world's rivers is widespread and increasing for the purpose of supplying electricity, water, and food for a growing population (Laize et al., 2014; Nilsson et al., 2005). At the same time, there is increasing recognition of the need to conserve riverine and riparian ecosystems and to provide recreational opportunities and other ecosystem services along rivers (Carlisle et al., 2011; González et al., 2017). Resolving this conflict requires improved understanding of the relationship between river flow and riverine ecosystem response, including the time scale of that response, so that water can be used efficiently for environmental purposes (Horne et al., 2017).

In North America, this conflict is most severe in the Colorado River Watershed, the principal water source for the Southwestern United States and much of Northwestern Mexico. Withdrawals from the Colorado River are so extensive that flows only occasionally reach the ocean, yet demand continues to rise, even as rising temperatures increase evaporative losses amid an ongoing multidecade drought (Wheeler et al., 2021). The Colorado River Watershed is also a focus of conservation activities to maintain the riverine ecosystem, endangered and threatened fish, and increasing recreational activities along the river and its tributaries. Decreasing peak and mean flows and decreasing flow variability are altering riparian vegetation, and these changes to vegetation have cascading influences on channel complexity, stability, and habitat for fish and other species (Butterfield et al., 2020; Grams et al., 2020). For this reason, the National Park Service and other agencies are now monitoring changes in riparian vegetation in response to flows (Auble et al., 1994, 2005; Perkins et al., 2018; Sankey et al., 2015; Scott & Friedman, 2018). The earliest long‐term riparian vegetation monitoring program in the Colorado River Basin began along the Gunnison River in 1990 (Auble et al., 1994). The current study examines 23 years of flow and vegetation data from this program.

The distribution of riparian vegetation along a floodplain cross section is strongly related to the hydrologic gradient, typically quantified as inundation duration (Auble et al., 1994, 2005; Jansson et al., 2019; Primack, 2000), inundation frequency (Friedman et al., 2006; Hupp & Osterkamp, 1985), or depth to water table (Camporeale & Ridolfi, 2006; Rains et al., 2004). Relations between plant occurrence and inundation duration can be combined with anticipated changes in flow duration to predict changes in the abundance or area occupied by a species or community (Auble et al., 2005; Primack, 2000; Toner & Keddy, 1997). For example, flow regulation without diversion tends to reduce peak flows while increasing minimum flows. This contraction of the hydrologic gradient narrows the zone subject to temporary inundation where riparian vegetation occurs (Auble et al., 1994; Jansson et al., 2019). Similarly, flow diversion decreases inundation durations and lowers water tables, reducing occurrence of hydrophytic species (Stromberg et al., 2007).

Zonation of species along the hydrologic gradient reflects intercorrelated gradients in fluvial disturbance intensity, frequency, and duration (Friedman et al., 1996; Hupp & Osterkamp, 1985; Menges & Waller, 1983), water, light, and nutrient availability (Araya et al., 2011), anoxia and drought (Winter, 2003), sediment particle size and organic matter content (Wilson & Keddy, 1985), propagule dispersal (Nilsson et al., 2010), and competition for water, light, and nutrients (Menges & Waller, 1983; Merritt et al., 2010; Wilson & Keddy, 1985). Because these factors are correlated and interdependent, their effects are difficult to distinguish using the most common research approach consisting of a gradient analysis based on data from a single point in time. This limits our ability to devise management interventions. Repeating observations over many years makes it possible to determine which of several species whose distribution is correlated to the hydrologic gradient will actually respond to a change in flow.

Analysis of plant traits allows examination of mechanisms controlling vegetation occurrence beyond what can be learned from individual species responses (Hough‐Snee et al., 2015; Lavorel & Garnier, 2002). The gradient of decreasing inundation duration away from a river often corresponds to increasing stem specific gravity, seed mass, plant height, longevity, ability to spread via rhizomes or stolons, total cover and biomass and a decrease in species richness (Friedman et al., 1996; Kyle & Leishman, 2009; McCoy‐Sulentic et al., 2017). High species richness often observed near a river is maintained by frequent fluvial disturbance and intermittent hypoxia, which prevent development of a dense canopy, allowing seedling establishment of many species. Higher on the bank, reduced disturbance allows tall, rhizomatous perennials to proliferate, increasing competition for light and moisture, which reduces opportunities for seedling establishment, leading to decreased species richness (Kotowski et al., 2010; Shipley et al., 1991). Reservoir operation typically reduces peak flows and increases low flows, allowing encroachment of dense vegetation toward the channel, which should narrow the zone occupied by ruderal species (Janssen et al., 2020; Tonkin et al., 2018).

Riparian ecosystems are strongly influenced by flow variation at scales ranging from minutes to decades or centuries (Friedman et al., 1996; Webb & Leake, 2006). Designing flows to promote a desired change in riparian vegetation requires an understanding of the time scale of the vegetation response. Addressing this question requires repeated observations of vegetation over decades in fixed plots of known inundation history, but such long‐term studies are rare (Sarneel et al., 2019; Winter, 2003). Over the last several decades, innovations in remote sensing and computational power have enabled analysis of factors controlling occurrence of riparian vegetation at regional and broader scales (McShane et al., 2015; Schneider et al., 2017), but rapid advances in the spatial scale of analysis have obscured slower advances at the temporal scale. Long‐term studies relating flow to changes in riparian vegetation are essential, even if they are initially limited in spatial scale. This study tracked vegetation in 133 permanent plots of known inundating discharge over 23 years to relate changes in species occurrence to short‐ and long‐term flow variation along the Gunnison River in the Upper Colorado River Basin in the United States. We investigated the time scale of the flow–vegetation relation controlling changes in vegetation between years and the long‐term effects of upstream reservoir construction on vegetation trends over the entire 23‐year period. We documented the movement of species up and down the hydrologic gradient and tested the hypotheses that (1) recent flows have a stronger influence on these temporal changes than flows in past decades and (2) the decrease in flood disturbance from flow regulation has led to a decrease in species richness and increases in mean plant height, the proportion of species that are perennials, and the proportion of species that have rhizomes.

## METHODS

### Study location

**FIGURE 1:**
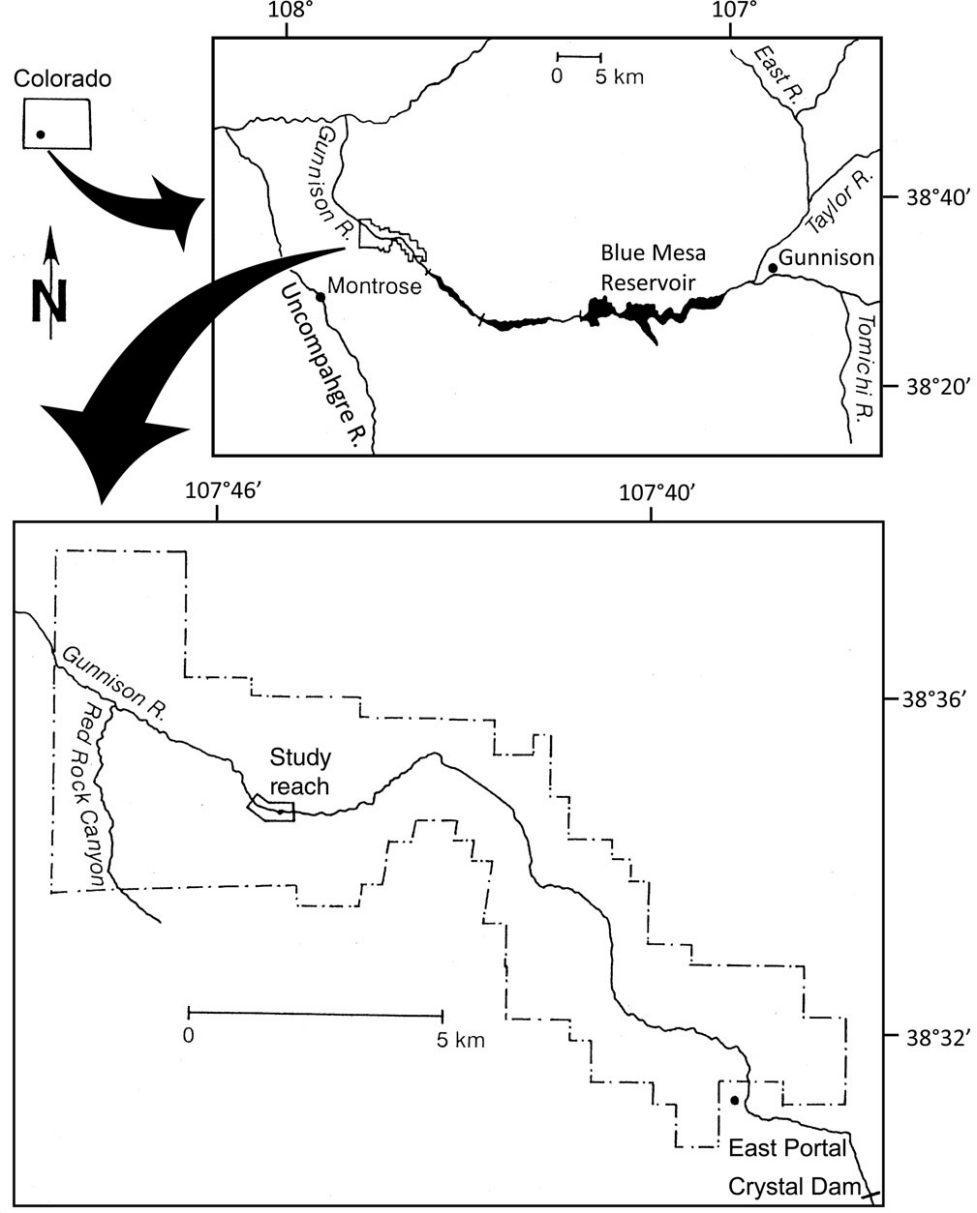
Location of study site along Gunnison River in Black Canyon of the Gunnison National Park, Montrose County, Colorado, in West Central United States. Figure modified from Friedman and Auble (1999). Flow in the Gunnison River is to the west.

The Gunnison River drains the western slope of the Rocky Mountains, flows for 77 km through Black Canyon, and joins the Colorado River in western Colorado. The study area is a 450‐m section of the Gunnison River within Black Canyon of the Gunnison National Park (Figure 1). Flow is strongly regulated by upstream reservoirs. The river is constrained within an extremely deep narrow canyon carved into metamorphic and igneous rocks (Dubinski & Wohl, 2007). Within the study site, the combined width of the floodplain and channel varies from 40 to 90 m, the gradient is 0.0128 m/m, and the elevation is 1707 m. The watershed area is ~10,000 km^2^. The average annual precipitation in the nearby town of Montrose at 1764 m elevation is 259 mm. (Colorado Climate Center, 2021). Peak discharge generally results from snowmelt in May and June. This section of the river consists of pool riffle sequences interrupted by rapids formed by tributary debris flows (Dubinski & Wohl, 2007; Elliott & Hammack, 2000). Riparian vegetation occurs on alluvial cobble and boulder bars with intermixed finer sediment. The vegetation is predominantly herbaceous with occasional *Acer negundo* (box elder) trees. The study reach is inaccessible to livestock.

**FIGURE 2:**
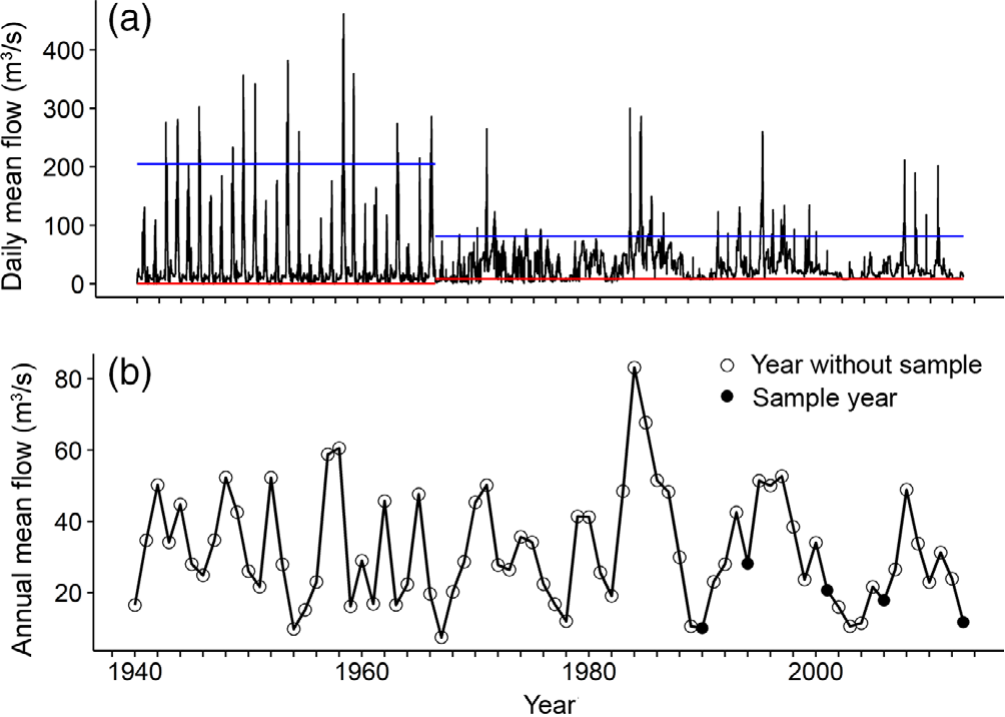
Flows at US Geological Survey Gage 09128000, Gunnison River below Gunnison Tunnel, Colorado, 14.5 km upstream of study site. (a) Daily mean flow. Blue and red lines indicate median annual maximum and minimum daily flows for preregulation period (1939–1965) and postregulation period (1966–2013). (b) Mean annual flow for year prior to sampling (18 July–17 July).

In 1934, there was little vegetation in the canyon bottom, probably because of the combined effects of alternating scouring floods and drought during very low flows (Warner & Walker, 1972). Between 1936 and 1976, four dams were constructed on the Gunnison and Taylor rivers upstream of the park for power generation and irrigation; the largest of these, with the greatest influence on flow, was Blue Mesa Dam (Figure 1), completed in 1965. Operation of the dams has reduced peak flows, raised low flows (Figure 2), and decreased sediment loads, limiting the movement of cobbles and boulders on the floodplain (Dubinski & Wohl, 2007; Elliott & Hammack, 2000) and enabling encroachment of vegetation (Friedman & Auble, 1999). The closest dam upstream of the park (Figure 1) removes hourly fluctuations in flow that can result from electricity generation.

### Vegetation sampling and characterization

In 1990, we established 133 rectangular plots, 1 × 2 m, oriented with the long axis parallel to flow at randomly selected locations within a floodplain area totaling 0.603 ha (Auble et al., 1994). We surveyed plot locations with a total station surveying instrument to allow plot relocation and quantification of plot inundation. We first sampled the plots on 18–31 July 1990. We planned to resample the plots in the same seasonal window every 4–5 years, although access and safety issues, mostly related to high flow, prevented sampling in some years. Plot resampling occurred on 29 July–1 August 1994, 20–22 July 2001, 22–24 July 2006, and 27–29 July 2013. The occurrence of all plant species in all plots was recorded in all 5 years. The present study is the first analysis of the data collected after 1990. Taxonomy follows the US Department of Agriculture (2020). We also recorded the proportion of each plot covered by living vegetation. To maintain consistency across years, at least two out of the three authors Friedman, Scott, and Shafroth led the vegetation crew in all five sample years, and a set of voucher specimens was maintained to help identify difficult plants. Plot data and photographs of the plots taken from consistent photo points in all five sample years are available in Friedman et al. (2022). For each species, we used information published in Ackerfield (2015) supplemented by the US Department of Agriculture (2020) to determine mean height (in decimeters), annual to perennial life span (0 for annuals, 0.5 for biennials, and 1 for perennials), and the presence or absence of rhizomes or stolons. For each plot, we used this information from the literature to calculate the mean height of species in the plot, the proportion of those species that were rhizomatous, and mean life span averaged across all 5 years and related these values to plot inundation duration averaged across years. We generated LOESS trendlines with 95% confidence intervals using the geom_smooth function in the R ggplot2 library.

To represent similarity in species composition of plots between years, we used the Sørensen index 2*a*/(2*a* + *b* + *c*), where *a* is the number of species common to both years, *b* is the number of species present in the first year but not the second, and *c* is the number of species present in the second but not the first. This index is suitable for data sets with many rare species (Hubálek, 1982). We calculated the Sørensen index for each successive pair of years in each plot and used the mean of all four pairs of years as the index of temporal variability for the plot.

### Modeling plant response to flow

To characterize the hydrologic gradient, we used a hydraulic model to determine the flow necessary to inundate each plot (inundating discharge), and we determined the proportion of days under water (inundation duration) using daily discharge measurements from a nearby stream gage (Auble et al., 1994, 2005). In 1990, we surveyed a sequence of nine hydraulic cross sections along the Gunnison River, including four within the study reach. We determined the relation between river flow and water surface elevation at each cross section using the HEC‐2 model (Hydrologic Engineering Center, 1990). We calibrated the hydraulic model using field observations of water level at all cross sections in 1990 at a wide range of flows: 9.5, 17.4, 21.7, 29.0, and 44.9 m^3^/s (Auble et al., 1994). The vegetation plots are in low‐gradient sections of river between rapids. The hydraulic cross sections are located so that each plot is between two cross sections with no intervening rapids. We used linear interpolation between cross sections to estimate the flow necessary to inundate each plot. River flow is measured 14.5 km upstream at US Geological Survey Gage 09128000, Gunnison River Below Gunnison Tunnel, in Montrose County, Colorado, located near East Portal (Figure 1). There are no dams, diversions, or important tributaries between the gage and the study reach. Because of confinement by the narrow canyon and sediment trapping and flow reduction from the dams just upstream, sediment movement along this river reach is low (Dubinski & Wohl, 2007), and plot elevations were assumed to be invariant across sample years. To check the validity of this assumption, we remeasured plot elevations in 2013 and found an elevation change from 1990 to 2013 of 0.088 m ± 0.11 m (mean ± SD) with a range of −0.35 to 0.59; 89% of plots had changed in elevation by an absolute value of 0.2 m or less. In comparison, the range in plot elevations relative to the river water surface is 1.6 m. In summary, mean changes in plot elevation were negligible, and only a small number of plots experienced a large enough change in elevation to substantially affect inundation duration.

To characterize the flow leading up to each sampling date, we used a 20‐year sliding period of record; for example, the period of record begins in WY1970 for the 1990 sample year and in WY1974 for the 1994 sample year. This sliding period was long enough to explore multidecade flow effects, allowed the same length of record to be analyzed for each sample year, and ensured that flow metrics calculated to characterize the earliest sample years were not influenced by the strongly different flows prior to the construction of Blue Mesa Dam in 1965. The sampling dates ranged from 18 July to 1 August across all survey years; therefore, 17 July was the last daily discharge used to calculate the flow record for each sample year.

Given that vegetation occurrence is strongly related to inundation, we expected that the hydrologic position occupied by a species in a given year would be related to recent flow. In other words, following increased flows, plants would be found in plots with higher inundating discharges. This argument implies that plants respond to a weighted version of the flow record in which recent flows count more than flows in the distant past, but how much more? In other words, we seek to know the time scale of the response of vegetation to changing flows. Our approach was to explore a range of weighted flow records and to select the one that best predicted the differences in species occurrence among sample years.

We represented the changing weights as an exponential decay process. In other words, the flow record for each sampling date was log‐weighted by recency to reduce the influence of flows farther in the past:

(1) $$w=e^{\left(\frac{t\left(\ln\left(0.5\right)\right)}{t_{\text{half}}}\right)}$$

where *w* is the weight applied to a particular day in the flow record ranging from 0 to 1, e is the base of the natural logarithm, 2.71828, *t* is the number of years prior to sampling (with days represented as fractional years), ln is the natural logarithm, and *t*_half_ is the number of years necessary to reduce the weight by half (Simmons, 1991).

We used linear regression to relate the median inundating discharge of all the plots containing a species in a given year to the mean weighted daily flow for the 20 years prior to sampling (below referred to as half‐life regressions). For each species we repeated the regression using 90 different half‐lives for the flow weights 0.1, 0.2, 0.3 … 5.0, 5.5, 6.0, 6.5 … 25 years. These half‐lives were more closely spaced at low values where the regression was more sensitive to half‐life variation. We selected the half‐life that maximized the variance explained by the regression (*R* ^2^). We used a two‐tailed *t*‐test to determine whether the slope of the regression was different from zero. For this analysis we considered only the 37 species that occurred in at least two plots in each of the five sample years. For species that produced significant half‐life regressions (*p* < 0.05), the half‐life for the species was used to weight the inundation duration for the logistic regression distribution model described in what follows. If the half‐life regression models found no significant half‐life for a species, the median half‐life from all the significant models (1.5 years) was used to weight the inundation duration in the logistic regression distribution model.

To estimate the optimum inundation duration of each species we performed logistic regression using a second‐order polynomial in the environmental variable (Auble et al., 2005; Jansson et al., 2019) on all 5 years of occurrence data using glm from the stats package in base R (R Development Core Team, 2019). Inundation duration, calculated using weighted flows as described previously, was cube‐root‐transformed prior to analysis to evenly distribute plots along the gradient and then back‐transformed for the presentation of results. Without this transformation, plots at the wet end of the inundation duration gradient would have had greater leverage than plots at the dry end. For each model we calculated the optimum inundation duration (the inundation duration with the highest modeled probability of occurrence for the species), the max‐rescaled Pseudo *R* ^2^ of Nagelkerke, and the likelihood ratio test statistic, distributed as χ^2^ with 2 df, for the null hypothesis that the explanatory variable *inundation duration* has a regression coefficient of 0. If the calculated optimum inundation duration was <0 (*n* = 3 species), it was reset to 0, and if the calculated optimum was >1 (*n* = 1 species), it was reset to 1. If the logistic regression was nonsignificant (*n* = 24 species) or produced a significant sigmoidal model with no optimum (*n* = 5 species), the optimum inundation duration was set to the median value of the plots where the species was present, using half‐life = 1.5 years.

### Analysis of temporal trends

We tested for temporal trends in the number of species per plot, mean height of species in the plot, proportion of those species that were rhizomatous, and their mean life span. Trends over time were calculated with a mixed linear regression model using plot as a random variable in a repeated‐measures design. *Year* and weighted mean daily *flow* (half‐life = 1.5) were both continuous fixed effects. Using this mixed model, we reported the within‐plot variance, which can be explained by *year* and *flow*, and the residual variance, which must be explained by other factors. For each of the fixed effects we reported the estimated slope, total change, and *p*‐value from the *t*‐test statistic for the null hypothesis that the coefficient of the explanatory variable was equal to zero. We carried out this analysis for all plots and for the subset of plots containing the invasive, highly competitive reed canary grass (*Phalaris arundinacea*) in all five sampling years. To identify species that increased or decreased in frequency of occurrence over the 23‐year period of study, we used a linear regression model with year as the predictor and number of occurrences as the response variable. A significant two‐tailed *t*‐test (*p* < 0.05) indicated the number of occurrences for that species increased or decreased over time.

## RESULTS

Dam operations have decreased the annual maximum daily flow from a median of 203 m^3^/s (1939–1965) to 81 m^3^/s (1966–2013) and increased the annual minimum daily flow from a median of 0.3 m^3^/s (1939–1965) to 8.6 m^3^/s (1966–2013; Figure 2). Over the same time, mean annual flows have changed little (from 33 to 31 m^3^/s). Flows are still dominated by montane snowmelt, and peak flows still usually occur in May and June. Annual mean flow in the sample years ranged from low to moderate (Figure 2 and Table 1).

**TABLE 1:** Comparison of peak, mean, and weighted mean flows for the five sample years

Year	Peak flow	Mean flow	Weighted flow
1990	25.94	10.13	23.88
1994	89.48	29.84	31.22
2001	56.92	21.05	29.97
2006	32.00	17.04	18.56
2013	20.42	11.52	21.96

*Note*: Peak flow is annual peak daily flow for year prior to sampling (18 July–17 July). Mean flow is mean daily flow for year prior to sampling (18 July–17 July). Weighted flow is weighted mean daily flow using a half‐life of 1.5 years for flow through 17 July of sampling year.

We identified 107 vascular plant species in our plots (Friedman et al., 2022). Vegetation was predominantly herbaceous. Herbs included 94 species (88%) and 5874 (96%) of the 6150 total occurrences. Trees, primarily *Acer negundo*, occurred in the plots, but only as seedlings or saplings under 2 m tall. Therefore, we set the height of all trees and shrubs to 20 dm. The number of species per plot ranged from 0 to 22 (mean ± SD = 9.2 ± 4.1). Vegetative cover ranged from 0% to 100% (mean ± SD = 53.9% ± 29%).

**FIGURE 3:**
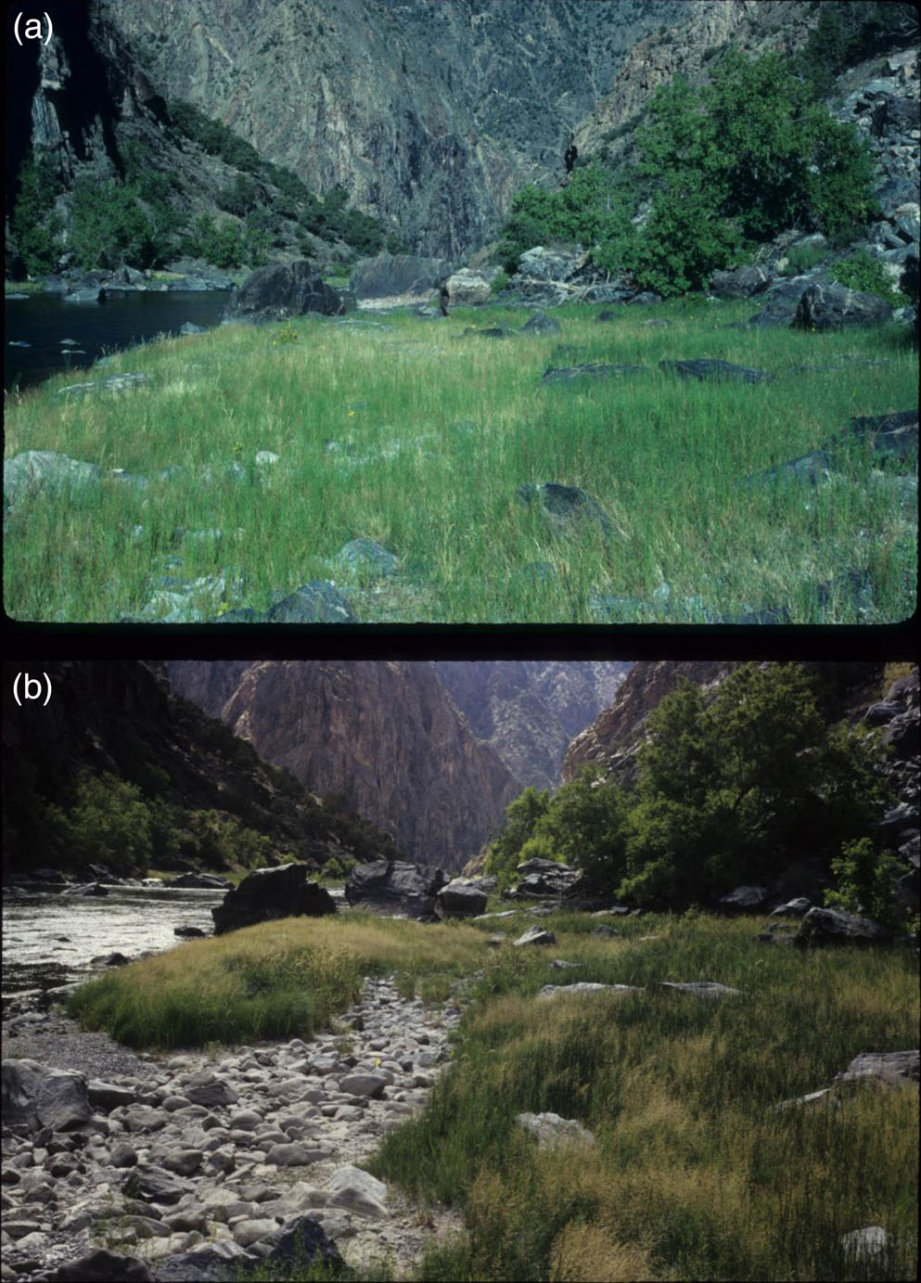
Matched photos showing vegetation change over time on one of five bars with permanent plots along Gunnison River in Black Canyon of the Gunnison National Park, Colorado. (a) 29 August 1990. (b) 2 August 1994. Moderately high flows in 1993 removed vegetation from the low‐lying area in the lower left.

**FIGURE 4:**
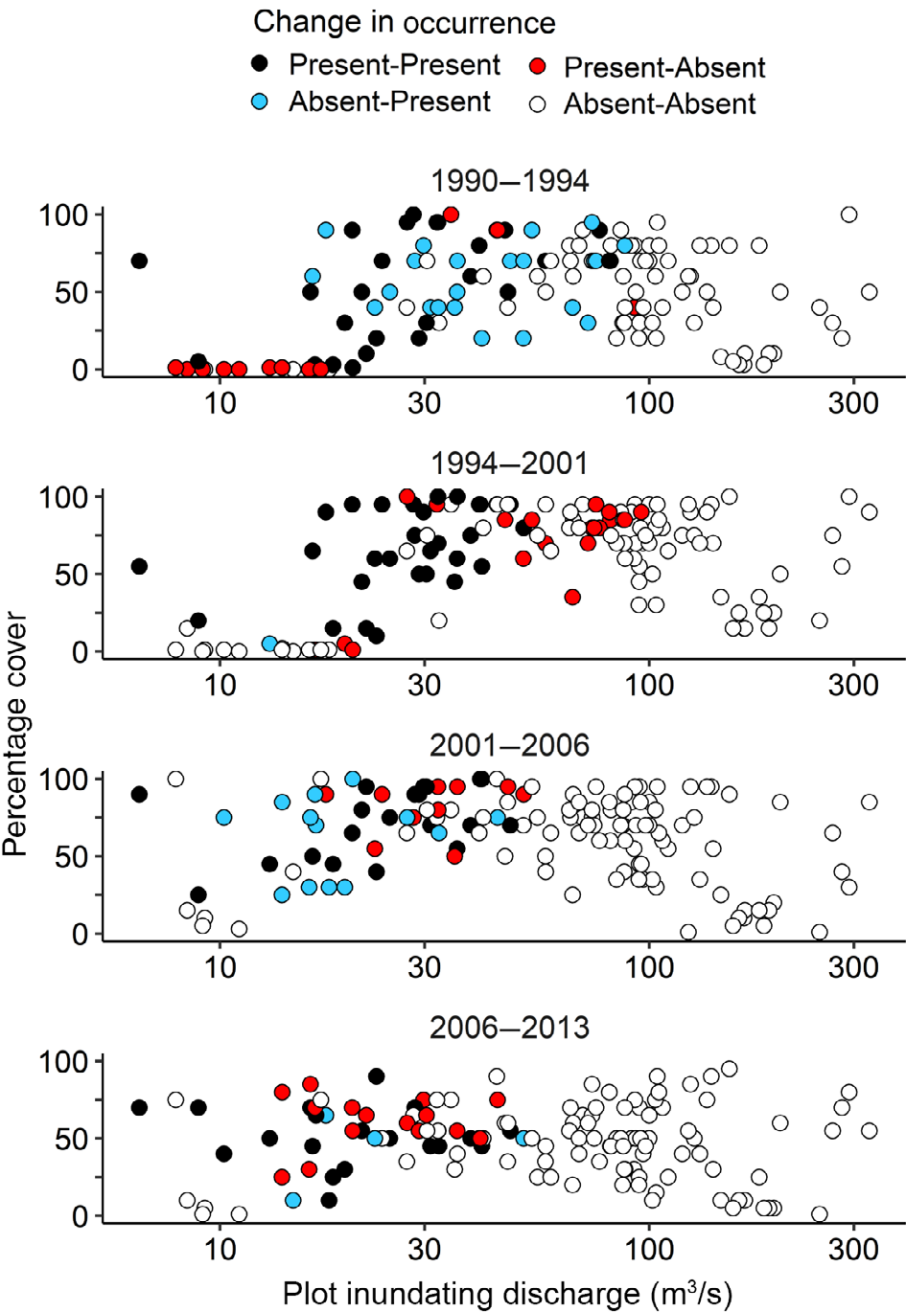
Change in occurrence of *Eleocharis palustris* between sample years as a function of plot inundating discharge and percentage cover in final year of each pair. Each dot is a plot, and each panel shows appearance and disappearance of *E. palustris* (colors) between the two sample years indicated above panel. The *x*‐axis is plotted on a log scale.

**FIGURE 5:**
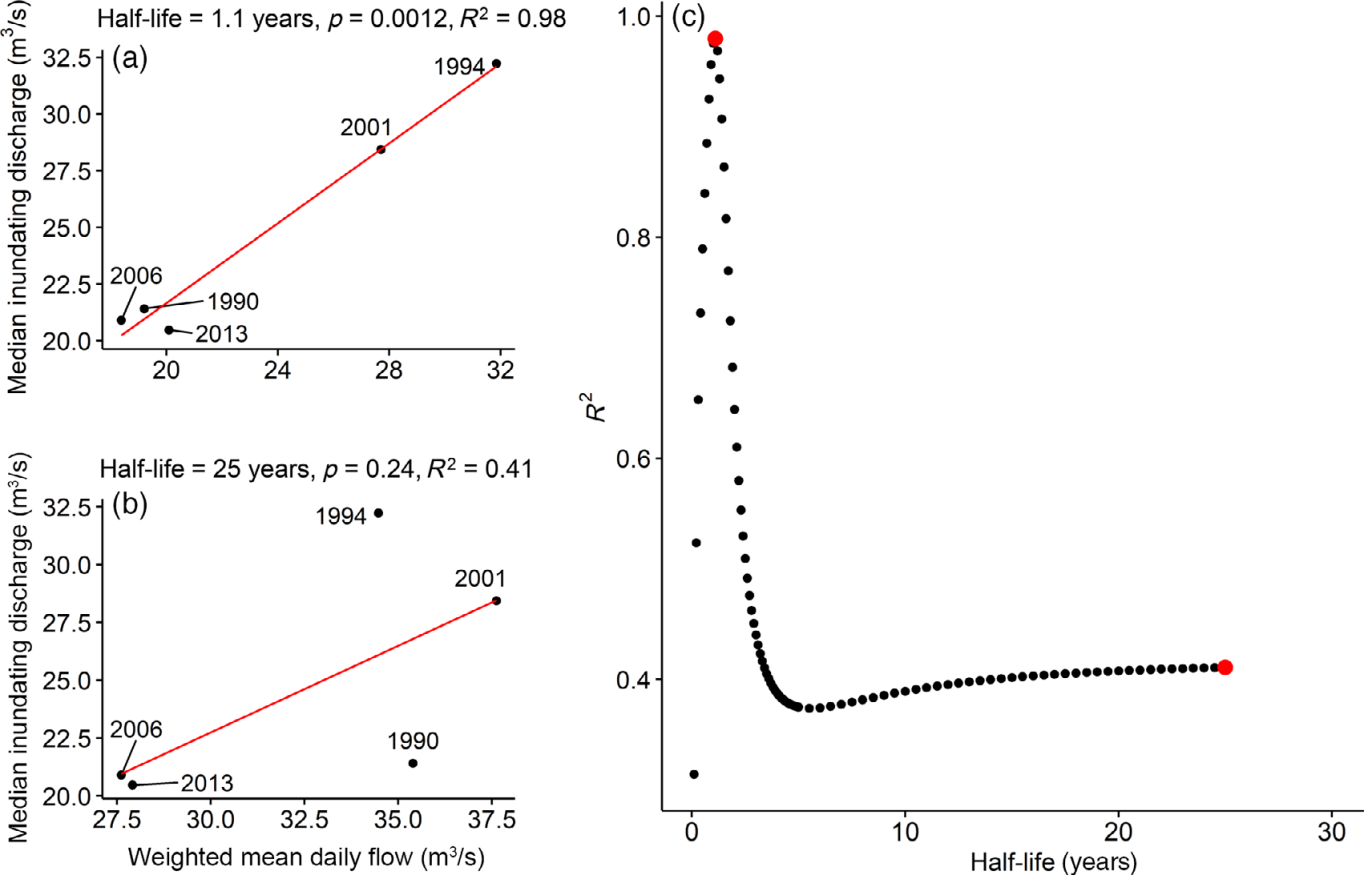
Linear regressions relating median inundating discharge of plots containing *Eleocharis palustris* to weighted mean daily flow prior to sample year (half‐life regressions). (a) Half‐life = 1.1 years. (b) Half‐life = 25 years. (c) *R* ^2^ as a function of half‐life for 90 linear regressions calculated as in (a) and (b). Each dot is a separate linear regression. The two red dots are the linear regressions shown in (a) and (b).

Plant species moved up and down the hydrologic gradient over time coincident with varying flows, resulting in changes in overall vegetative cover, especially near the channel (Figure 3). We use *Eleocharis palustris* to illustrate our analyses of plant responses to flow. This species is abundant in frequently inundated locations. In 1990, after 2 years of low flow, *Eleocharis* was entirely restricted to surfaces with inundating discharge below 100 m^3^/s with only five occurrences above 50 m^3^/s (Figure 4). In 1994, after 4 years of moderate (increased) flows, it had increased occurrence in higher plots but had been removed, along with almost all other species, from the lowest plots, as indicated by low plot vegetative cover (Figure 4). From 1994 to 2001 *Eleocharis* was removed from all plots with inundating discharge above about 50 m^3^/s, but not plots with lower inundating discharge. The location of *Eleocharis* along the inundating discharge gradient is positively correlated with flow (Figure 5). The relation between median inundating discharge of *Eleocharis* occurrences and weighted mean daily flow is strongest when the weighting half‐life = 1.1 years (Figure 5c), in other words, when the flow 1.1 years before sampling is weighted half as strongly as the flow at the time of sampling.

**FIGURE 6:**
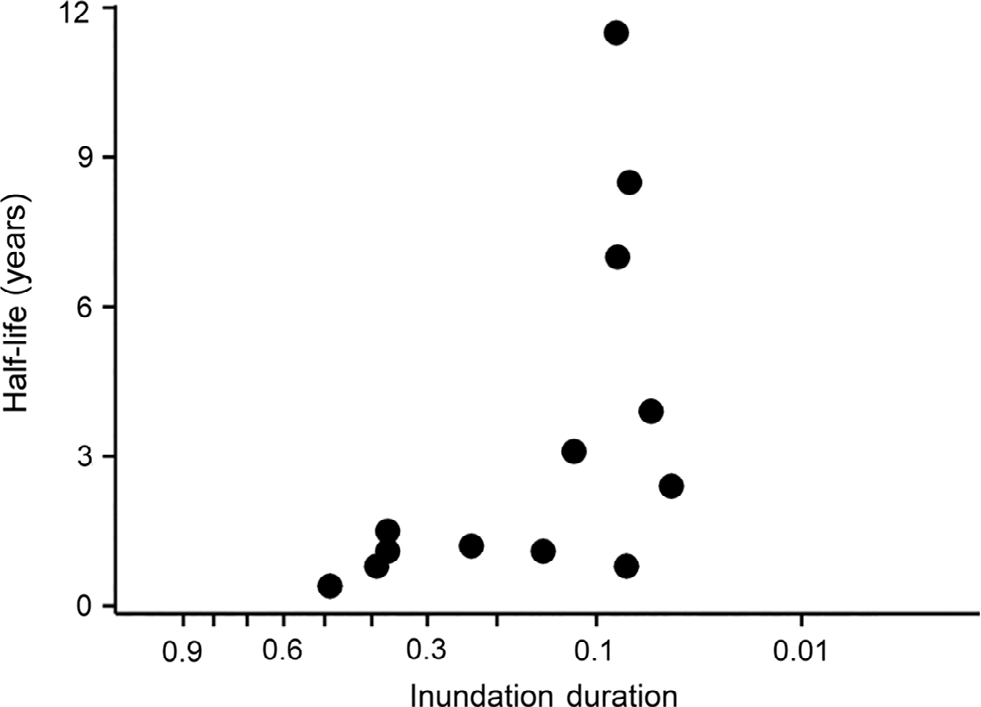
Species half‐life in years as a function of species optimum inundation duration (plotted on cube‐root scale) at Black Canyon of the Gunnison National Park, Colorado. Inundation duration ranges from 1 (always inundated) to 0 (never inundated). Only species with significant half‐lives are shown.

The half‐life regression illustrated for *Eleocharis* in Figure 5 was repeated for all species with at least two occurrences in all five sample years (Table 2). Thirteen species, including *Eleocharis*, showed a significant positive relation between median inundating discharge and weighted mean daily flow, indicating that these species moved to higher ground in response to increased flow and to lower ground in response to decreasing flow. All these species had an optimum inundation duration above 0.05; in other words, they were all from the hydric or mesic part of the hydrologic gradient. No species had a significant negative relation between inundating discharge and flow. Half‐lives for the weighting of flow in significant models ranged from 0.4 to 11.5 years with a median of 1.5 (Table 2 and Figure 6), indicating that most species respond most strongly to flows in the last year or two. Half‐lives over 2 years were mostly found for rhizomatous perennial species with a moderate optimum inundation duration (around 0.1; Figure 6), such as *Asclepias speciosa* (showy milkweed), *Carex pellita* (woolly sedge), and *Equisetum hyemale* (scouring horsetail, Table 2). The slope of half‐life regressions ranged from 0.45 to 3.98, with a median of 1.73 and a mean value of 1.68 (Table 2); in other words, an increase in the weighted mean daily flow of 1.0 m^3^/s caused species to move up the inundating discharge gradient by 1.68 m^3^/s. Slope was not correlated with half‐life (*p* = 0.995). Seventy species had significant logistic regression models relating occurrence to inundation duration, including 41 of 44 species with more than 25 occurrences (Supporting Data). The inundation duration with the highest probability of occurrence for a given species (optimum inundation duration) had a median of 0.19.

**TABLE 2:** Abundance, traits, and results of half‐life regressions and logistic regressions for all species with at least two occurrences in each sample year

Genus species	No. occurrences	Height (dm)	Life span	Rhizomes	Median inundating discharge (m^3^/s)	Half‐life regressions				Logistic regressions		
						Half‐life (years)	Slope	*p*	*R* ^2^	Optimum inundation duration	*p*	*R* ^2^
*Acer negundo*	108	20	P	No	36	6.0	−1.1	0.126	0.60	0.302	**<0.001**	0.09
*Agrostis gigantea*	449	7	P	Yes	51	1.1	2.0	**0.021**	0.87	0.148	**<0.001**	0.24
*Apocynum cannabinum*	204	7.5	P	Yes	59	0.8	1.9	**0.048**	0.78	0.078	**0.005**	0.02
*Aristida purpurea*	25	5.5	P	No	167	1.9	0.03	0.388	0.25	0.000	**<0.001**	0.40
*Artemisia ludoviciana*	106	6	P	Yes	83	0.5	3.0	0.124	0.60	0.000	**0.047**	0.02
*Asclepias speciosa*	178	8	P	Yes	75	3.9	1.7	**0.016**	0.89	0.063	**<0.001**	0.16
*Bromus arvensis*	41	4.5	A	No	127	1.5	5.5	0.230	0.43	0.000	**<0.001**	0.13
*Bromus tectorum*	121	4.5	A	No	147	1.7	3.6	0.114	0.62	0.003	**<0.001**	0.44
*Carex nebrascensis*	102	7	P	Yes	81	11.5	1.8	**0.029**	0.84	0.085	**<0.001**	0.07
*Carex pellita*	339	6.5	P	Yes	69	3.1	1.8	**0.009**	0.92	0.119	**<0.001**	0.14
*Cirsium arvense*	77	11.5	P	Yes	91	2.4	4.0	**0.002**	0.97	0.052	**<0.001**	0.05
*Convolvulus arvensis*	51	0.5	P	Yes	100	7.0	−2.5	0.150	0.55	0.000	**<0.001**	0.15
*Eleocharis palustris*	180	7.5	P	Yes	25	1.1	0.9	**0.001**	0.98	0.370	**<0.001**	0.49
*Elymus repens*	136	7.5	P	Yes	74	7.0	1.8	**0.009**	0.93	0.084	**<0.001**	0.20
*Epilobium ciliatum*	94	10.25	P	No	22	0.8	1.3	**0.009**	0.93	0.390	**<0.001**	0.33
*Equisetum arvense*	90	3	P	Yes	24	0.9	1.0	0.079	0.70	0.433	**<0.001**	0.22
*Equisetum hyemale*	511	6	P	Yes	72	8.5	0.8	**0.015**	0.90	0.076	**<0.001**	0.22
*Euthamia occidentalis*	472	12	P	Yes	51	1.0	0.6	0.353	0.29	0.171	**<0.001**	0.27
*Heterotheca villosa*	61	4	P	No	162	0.1	0.8	0.179	0.50	0.002	**<0.001**	0.25
*Juncus arcticus*	128	6	P	Yes	76	4.6	−0.5	0.677	0.07	0.045	**<0.001**	0.17
*Juncus dudleyi*	38	6	P	No	28	0.9	1.3	0.109	0.63	0.299	**<0.001**	0.15
*Melilotus officinalis*	147	10	AB	No	41	0.1	1.2	0.139	0.57	0.157	**<0.001**	0.05
*Mentha arvensis*	85	4	P	Yes	40	0.1	0.3	0.363	0.28	0.178	**<0.001**	0.12
*Muhlenbergia racemosa*	173	7	P	Yes	70	0.9	1.8	0.314	0.33	0.086	**<0.001**	0.07
*Phalaris arundinacea*	362	12.5	P	Yes	41	0.9	0.9	0.152	0.55	0.206	**<0.001**	0.22
*Plantago major*	49	3	P	No	22	1.5	1.7	**0.006**	0.94	0.369	**<0.001**	0.30
*Poa palustris*	181	7.25	P	Yes	32	1.2	1.7	**0.040**	0.80	0.234	**<0.001**	0.22
*Poa pratensis*	396	3.75	P	Yes	77	1.0	1.2	0.076	0.70	0.045	**<0.001**	0.31
*Ranunculus cymbalaria*	42	1.6	P	Yes	22	1.3	1.2	0.328	0.31	0.381	**<0.001**	0.19
*Rumex sp*.	69	NA	P	NA	31	6.0	1.0	0.160	0.54	0.304	**<0.001**	0.11
*Salix exigua*	74	20	P	Yes	72	8.0	2.8	0.162	0.53	0.042	0.445	0.01
*Sporobolus cryptandrus*	65	6.5	P	No	166	6.0	1.0	0.144	0.56	0.000	**<0.001**	0.42
*Stachys pilosa*	35	5.75	P	Yes	70	0.5	1.6	0.249	0.41	0.037	**<0.001**	0.08
*Symphyotrichum lanceolatum*	81	4.75	P	Yes	66	0.7	−0.6	0.068	0.72	0.065	**<0.001**	0.09
*Toxicodendron rydbergii*	34	11.5	P	Yes	96	19.5	0.6	0.137	0.58	0.012	**<0.001**	0.12
*Trifolium repens*	60	2.15	P	Yes	31	0.1	0.8	0.250	0.40	0.271	**<0.001**	0.14
*Veronica anagallis‐aquatica*	49	3.5	P	Yes	16	0.4	0.4	**0.017**	0.89	0.489	**<0.001**	0.29

*Note*: The complete species list and plot data are available in Friedman et al. (2022). Taxonomic nomenclature follows US Department of Agriculture (2020). Under life span, P = perennial, B = biennial, A = annual. Data for height, life span, and rhizomes are from Ackerfield (2015) supplemented by US Department of Agriculture (2020). Significant *p*‐values (*p* < 0.05) are highlighted in bold. Optimum inundation durations are calculated using flows weighted for recency, as described in *Methods*. The *R* ^2^ values under logistic regression are the max‐rescaled pseudo‐*R* ^2^ of Nagelkerke.

**FIGURE 7:**
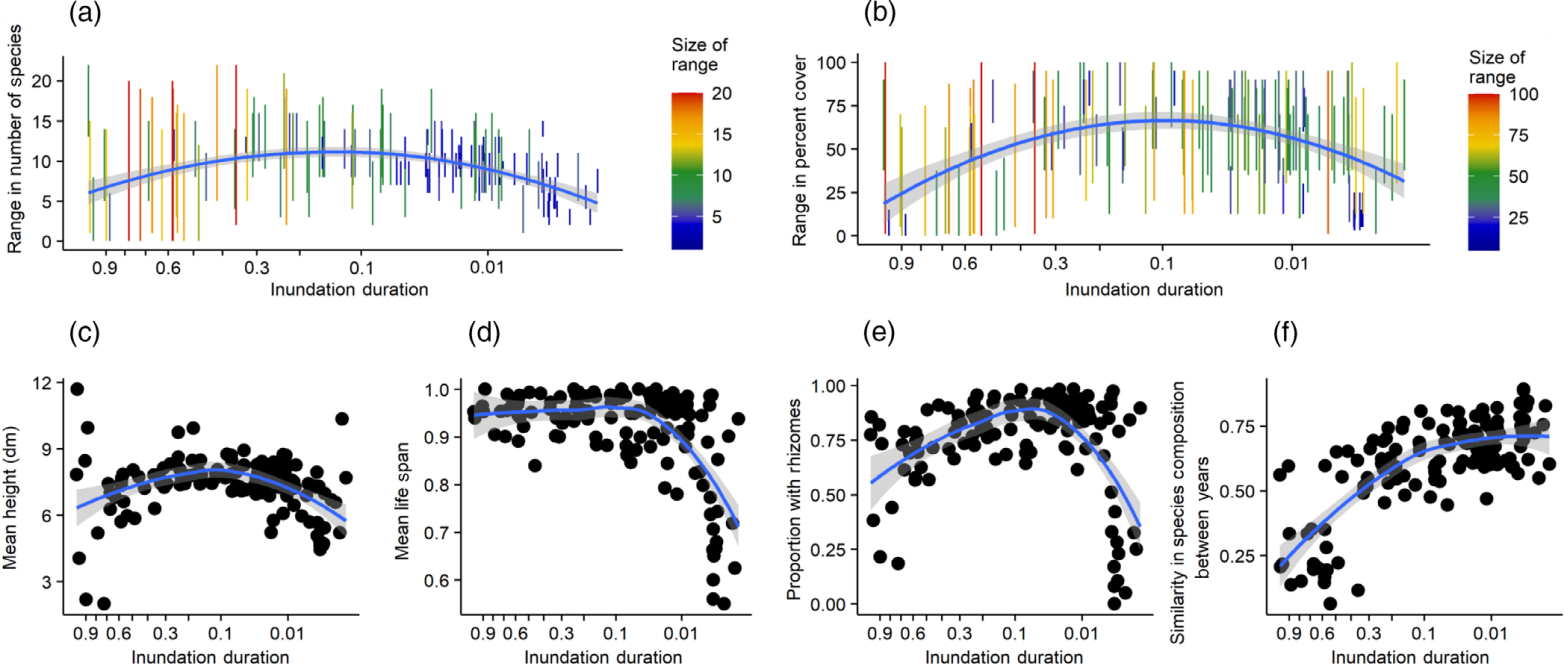
Plot characteristics at Black Canyon of the Gunnison National Park, Colorado. In all graphs, the *x*‐axis is inundation duration calculated with a weight of 1.5 years, averaged across the five sample years and plotted on cube‐root scale. Inundation duration ranges from 1 (always inundated) to 0 (never inundated). The blue line and gray shading are mean and 95% confidence interval. (a) Range in number of species per plot over five sample years. The vertical bar extends from lowest to highest observed number of species in plot, and bar color is scaled to its length. (b) Range in percentage cover of plot over five sample years. (c) Mean plant height for each plot averaged across all 5 years. (d) Mean life span for species in each plot (annual = 0, biennial = 0.5, perennial = 1) averaged across all 5 years. (f) Similarity in species composition (Sørensen index) within plots and between years.

Plot species richness, vegetative cover, mean plant height, and proportion of plants with rhizomes all had higher values in the middle of the inundation duration gradient than at either extreme (Figure 7). Mean life span had a similar pattern but lacked a decrease at high inundation durations. Plots at the wet end of the gradient had greater variation between years in species richness (Figure 7a) and, to a lesser extent, percentage cover (Figure 7b), reflecting the stronger influence of fluvial disturbance. For the same reason, plot similarity between years strongly increased with decreasing inundation duration (Figure 7f).

Over the 23‐year study period, rhizomatous perennial species increased relative to nonrhizomatous shorter‐lived species more dependent upon physical disturbance for propagation. Considering all plots, the mean life span and proportion of species with rhizomes increased, and the mean number of species per plot decreased by 1.97 species, even while accounting for the effect of flow variation (Table 3). Consistent with this trend, the five species that increased in occurrence over this period were all rhizomatous perennials, whereas the six decreasing species included only three perennials and only one with rhizomes (Table 4). The second most abundant of the increasing species is *Phalaris arundinacea* (reed canary grass), an invasive, tall rhizomatous perennial. Considering only plots in which *Phalaris arundinacea* was present in all sample years, the mean number of species per plot decreased even more strongly, by 5.02 species (Table 3). At the annual time scale, low flows, especially in 1990 and 2006, were associated with more species per plot, a shorter mean life span and lower proportion of rhizomatous species (Table 3).

**TABLE 3:** Results of mixed linear regression models testing whether vegetation in plots has changed over time or in response to flow variation

Model	Random‐effect *plot*		Fixed‐effect *year*			Fixed‐effect *flow*		
	Within plot variance	Residual variance	Slope	Total change (over 23 years)	*p*	Slope	Total change (across 12.7 m^3^/s)	*p*
All plots								
Number of species	6.060	10.460	−0.086	−1.978	**<0.001**	−0.201	−2.552	**<0.001**
Mean height (dm)	1.318	2.101	−0.013	−0.291	0.111	−0.024	−0.311	0.076
Mean life span	0.008	0.006	0.002	0.054	**<0.001**	0.003	0.035	**<0.001**
Proportion with rhizomes	0.047	0.024	0.005	0.124	**<0.001**	0.003	0.036	0.054
Plots with *Phalaris arundinacea* present in all 5 years								
Number of species	2.134	7.488	−0.218	−5.014	**<0.001**	−0.225	−2.857	**<0.001**
Mean height	0.094	0.599	−0.019	−0.448	**0.021**	−0.009	−0.109	0.619
Mean life span	0.0004	0.002	0.002	0.045	**<0.001**	0.002	0.02	0.055
Proportion with rhizomes	0.003	0.011	0.007	0.16	**<0.001**	0.005	0.069	**0.006**

*Note*: In these models, *plot* is a random variable, while *year* and *flow* are fixed effects. Plot characteristics examined are number of species, mean species height, mean species life span (annual = 0, biennial = 0.5, perennial = 1), and proportion of species with rhizomes. *Flow* is the weighted mean daily flow calculated with a half‐life of 1.5 years. Models were run first for all plots (133 plots) and then just for those plots with *Phalaris arundinacea* in all 5 years (34 plots). Significant *p* values (*p* < 0.05) are highlighted in bold.

**TABLE 4:** Characteristics of species increasing and decreasing over the 23‐year study

Genus species	Slope	*R* ^2^	*p*	Life span	Rhizomes	Mean height (dm)	No. occurrences	Optimum inundation duration
Increasing species								
*Asclepias speciosa*	2.0	0.86	0.023	P	Yes	8	178	0.063
*Phalaris arundinacea*	1.9	0.92	0.010	P	Yes	12.5	362	0.206
*Apocynum cannabinum*	1.2	0.96	0.003	P	Yes	7.5	204	0.078
*Equisetum hyemale*	0.9	0.82	0.036	P	Yes	6	511	0.076
*Carex praegracilis*	0.2	0.80	0.042	P	Yes	5	15	0.091
Decreasing species								
*Echinochloa crus‐galli*	−0.1	0.78	0.047	A	No	11	5	0.450
*Alopecurus aequalis*	−0.2	0.79	0.045	AP	No	4	9	0.557
*Tamarix ramosissima*	−0.3	0.92	0.011	P	No	20	18	0.040
*Verbena bracteata*	−0.7	0.78	0.049	ABP	No	3	29	0.179
*Acer negundo*	−1.3	0.87	0.021	P	No	20	108	0.302
*Agrostis gigantea*	−1.6	0.80	0.039	P	Yes	7	449	0.148

*Note*: Taxonomic nomenclature follows US Department of Agriculture (2020). Under life span, P = perennial, B = biennial, A = annual. Data for height, life span, and rhizomes are from Ackerfield (2015) supplemented by US Department of Agriculture (2020).

## DISCUSSION

Our results confirm those of other studies showing that riparian plant species are distinctly arranged along the hydrologic gradient (Auble et al., 1994, 2005; Camporeale & Ridolfi, 2006; Friedman et al., 2006; Hupp & Osterkamp, 1985; Jansson et al., 2019; Primack, 2000; Rains et al., 2004). More importantly, we found that plants moved rapidly up and down the hydrologic gradient in response to changing flows. We conclude that a half‐life of 1.5 years is a reasonable assumption for calculating flow relations for most herbaceous species in this study. This means that a long flow record may not be necessary to characterize the hydrologic relations of many riparian plants. The exceptions are some rhizomatous tall perennials, such as *Asclepias speciosa* and *Equisetum hyemale*, which can persist in the absence of disturbance and show relatively small changes between years. The slopes of the half‐life regressions show how far a species moved up or down the hydrologic gradient in response to changing discharge. These slopes are generally >1; in other words, when weighted mean daily flow increased by 1 m^3^/s, the median inundating discharge of plots including a species tended to increase by more than 1 m^3^/s, demonstrating a strong response of vegetation to changing flow.

Though 70 species had significant logistic regressions, only 13 species had significant half‐life regressions. This difference reflects the small sample size (*n* = 5 sample years) for the half‐life regressions as well as the stronger evidence of flow influence they required for a significant result. Logistic regression returned a significant result for any species with a strong pattern of occurrence against the inundation duration gradient, regardless of whether the species responded to changes in flow from one sample year to the next. In contrast, a significant half‐life regression indicates that the species moved up and down the bank between sample years in concert with changing flows, an important criterion of success for the design of flow releases from reservoirs to affect riparian vegetation.

Plot vegetative cover, mean plant height, and proportion of species with rhizomes all peak at moderate inundation durations. High vegetative cover and the dominance of tall, rhizomatous perennial plants like *Phalaris arundinacea* and *Euthamia occidentalis* (western goldentop) indicate strong competition for resources and reduced seedling establishment opportunities at moderate inundation durations. At higher inundation durations more frequent disturbance reduces vegetative cover in some years, allowing the establishment of ruderal disturbance‐dependent species like *Plantago major* (common plantain) and flood‐tolerant perennial species of lower stature like *Eleocharis palustris* and *Veronica anagallis‐aquatica* (water speedwell). At low inundation durations, dry conditions limit vegetative cover, allowing the occurrence of short ruderal drought avoiders like *Bromus tectorum* (cheatgrass) and *B. arvensis* (field brome) and perennial drought tolerators like *Heterotheca villosa* (hairy false goldenaster).

The influence of flow variability on riparian vegetation decreased with decreasing inundation duration. Plot vegetation similarity between years increased with decreasing inundation duration, species richness was most variable in plots with high inundation durations (above 0.2), and no species with an optimal inundation duration below 0.05 had a significant half‐life regression. These patterns reflect the decreased intensity of flood disturbance at the dry end of the hydrologic gradient. Similarly, along the Colorado River in the Grand Canyon, the influence of river flow on riparian vegetation cover decreased with decreasing inundation duration (Sankey et al., 2015). Our results are also consistent with the transplant experiments of Sarneel et al. (2019) along the Vindel River in Sweden, where species occurrence was limited near the channel by inundation and far above the channel by competition.

Our observed trends in plot‐based vegetation characteristics along the inundation duration gradient are consistent with other trait‐based gradient analyses and add to them by extending the hydrologic gradient up into the zone of water scarcity and by considering variation over time. From high to moderate inundation durations we saw trends of increasing vegetative cover, mean plant height, and proportion of species with rhizomes. These results confirm the observations of earlier studies (Kyle & Leishman, 2009; McCoy‐Sulentic et al., 2017; Shipley et al., 1991), but our observations extended further along the inundation duration gradient in a dry climate to locations of water scarcity, where competitive species gave way to small‐statured herbaceous stress tolerators and stress avoiders. This resulted in a curvilinear relation of competitiveness and plant cover to inundation duration, in contrast to the linear relations seen in other studies where the climate was not as dry (Shipley et al., 1991) or where the stress tolerators on dry sites were taller shrubs (McCoy‐Sulentic et al., 2017). By observing our plots multiple times in varying flows, we were able to document how the relative abundance of disturbance‐dependent and stress‐tolerant species at the wet end of the hydraulic gradient corresponded to greater temporal variability in plot species composition, vegetative cover, and species richness. These observations directly document the role of frequent disturbance in shaping the riparian plant community. The regional riparian flora consists mostly of species distributed broadly over Western North America (McShane et al., 2015), facilitating widespread application of our results.

Over the 23 years of this study, the mean life span and proportion of species with rhizomes increased and the mean number of species per plot decreased. The decrease in species richness was especially strong in plots occupied by the competitive invasive *Phalaris arundinacea*. Species increasing in occurrence over time were all rhizomatous perennials, whereas declining species tended to be shorter lived and nonrhizomatous. These changes are consistent with the decrease in the proportion of the canyon bottom annually cleared of vegetation by flood disturbance (Friedman & Auble, 1999) resulting from the decrease in peak flows associated with upstream reservoir construction, especially the completion of Blue Mesa Dam in 1965. This trend likely began immediately after dam construction and was ongoing in 2013, 48 years later, demonstrating a long‐term response of vegetation to flow regulation (Stromberg et al., 2012). The dominance of the vegetation at this site by rhizomatous perennials in all sample years demonstrates that by the beginning of our study, the abundance of species requiring frequent disturbance for persistence had already been depressed by flow regulation. In contrast, the nearby lightly regulated Yampa River, Colorado, experiences frequent flooding and greater channel movement, producing a large area of young fluvial surfaces, fostering many ruderal species (Merritt & Cooper, 2000; Scott & Friedman, 2018). A vegetative comparison of the Yampa River and strongly regulated Green River in northwestern Colorado showed that species richness was 40% higher on the less regulated river and decreased with increasing surface age on both rivers (Uowolo et al., 2005). Increases in competitive species following flow regulation have been observed and predicted elsewhere (Janssen et al., 2020; Tonkin et al., 2018). The long‐term decrease in riparian species richness associated with decreasing flow variability in the Upper Colorado River system is consistent with the inverse observation by Garssen et al. (2014) that an increase in flood frequency or severity in an arid or semiarid setting generally leads to an increase in riparian species richness.

The response of vegetation along the Gunnison River to changing flows differs across scales. At the multidecade scale, decreased peak flows and increased base flows caused by reservoir operations have allowed increases in rhizomatous perennial species. At the annual scale, low‐flow years are associated with increased species per plot and decreased mean life span and proportion of species with rhizomes. This is because low flows allow the establishment of ruderal species at low inundating discharges in the short term (Friedman et al., 1996). Over the longer term, drought stress and chronic reduction in flood disturbance promote the replacement of these ruderal species by more competitive species in a broad range of inundating discharges.

Both our half‐life and logistic regressions can be used to predict how plants move up or down the hydrologic gradient and change in area in response to changes in flow (Auble et al., 1994; Jansson et al., 2019). On the other hand, these relations do not calculate the area cleared by a particular flow (Friedman & Auble, 1999), nor do they model competitive exclusion as a function of time since surface formation. Models directly considering such transient effects (Diehl et al., 2018; Keddy & Campbell, 2020; Merritt et al., 2010) would be useful to predict the future abundance of flood‐dependent species in response to changes in flow regulation.

The assumption that plant occurrence is in equilibrium with flow fails for long‐lived species whose size and hydrologic position vary strongly with age. For example, seedlings of large woody species like *Acer negundo* and *Salix exigua* (narrowleaf willow) are found at different hydrologic positions from one year to the next in response to flow variation in the year of germination, but long‐term survival tends to be limited to surfaces with low inundation duration that are relatively safe from fluvial disturbance (DeWine & Cooper, 2007; Scott et al., 1997; Shafroth et al., 2010). Another abundant species in the region that is uncommon at this site but likely to exhibit similar patterns is *Populus fremontii* (Fremont cottonwood; Scott & Friedman, 2018). Given that the seedlings and adults of these species tend to occupy different hydrologic positions, it is not surprising that *Salix exigua* was one of the few common species that did not have a significant logistic regression model relating occurrence to inundation duration. In addition, the small plots used in this study (1 × 2 m) captured the seedlings and saplings of shrubs and trees, but rarely adults, precluding characterization of the hydrologic position occupied by these species.

This study is one of the first to use multiple repeat observations of riparian vegetation to examine the effects of changes in river flow over many years. The limited number of plots and resample years necessitated the equilibrium approach taken here and prevented the development of models that included other factors influencing vegetation change. Our inability to safely sample in high‐flow years reduced the accuracy of our analysis by reducing the range of conditions observed.

The design of this study constitutes an early prototype of the National Park Service Big Rivers Monitoring Program, which now includes annual resampling of the vegetation and elevation of permanent plots along many rivers in the Colorado River Basin and beyond (Perkins et al., 2018). Our results provide an analysis approach and a set of testable hypotheses for this and other programs designed to relate changing riparian vegetation to flow; for example, (1) herbaceous vegetation responds to flow changes on a 1.5‐year time scale and (2) at the decadal time scale, flow stabilization results in increased cover of rhizomatous perennials and decreases in other species. Finally, our results inform the design of reservoir releases to favor some species over others, to promote species richness, or to change the location of species within the riparian zone.

## CONFLICT OF INTEREST

The authors declare no conflict ofinterest.

## Data Availability

Data (Friedman et al., 2022) are available from USGS ScienceBase at https://doi.org/10.5066/P91BEXPC.
